# Integrating the social sciences into the COVID-19 response in Alberta, Canada

**DOI:** 10.1136/bmjgh-2020-002672

**Published:** 2020-07-27

**Authors:** Myles Leslie, Raad Fadaak, Jan Davies, Johanna Blaak, PG Forest, Lee Green, John Conly

**Affiliations:** 1 School of Public Policy, University of Calgary, Calgary, Alberta, Canada; 2 W21C Research and Innovation Centre, University of Calgary, Calgary, Alberta, Canada; 3 Cumming School of Medicine, University of Calgary, Calgary, Alberta, Canada; 4 Family Medicine, University of Alberta, Edmonton, Alberta, Canada; 5 Infection Prevention and Control, Alberta Health Services, Calgary, Alberta, Canada

**Keywords:** health systems, health policy, health services research, public health, epidemiology

## Abstract

This paper outlines the rapid integration of social scientists into a Canadian province’s COVID-19 response. We describe the motivating theory, deployment and initial outcomes of our team of Organisational Sociologist ethnographers, Human Factors experts and Infection Prevention and Control clinicians focused on understanding and improving Alberta’s responsiveness to the pandemic. Specifically, that interdisciplinary team is working alongside acute and primary care personnel, as well as public health leaders to deliver ‘situated interventions’ that flow from studying communications, interpretations and implementations across responding organisations. Acting in real time, the team is providing critical insights on policy communication and implementation to targeted members of the health system. Using our rapid and ongoing deployment as a case study of social science techniques applied to a pandemic, we describe how other health systems might leverage social science to improve their preparations and communications.

**Summary box**

- Calls for the integration of social scientists into pandemic preparedness and responses have increased in recent years.
- This paper describes the theoretical foundations, deployment and outcomes of an interdisciplinary team of social scientists, Human Factors experts and clinicians who responded to the COVID-19 pandemic in Alberta, Canada.
- Our team delivered ‘situated interventions’ that combined observations with recommendations in real-time as we worked alongside pandemic responders.
- The team focused on improving communications, providing cross-organisational awareness, and bolstering capacity to interpret and implement policy on the front lines of acute and primary care.
- Operational approvals and access within health system organisations may present significant obstacles for social scientists working to rapidly respond during pandemics. These approvals are often based on clinical research or clinical trial methodologies rather than social scientific ones.
- By positioning themselves as observers across many parts of a given health system, social scientists are uniquely equipped to document, analyse and improve that system’s emergency response.

## Introduction

For over two decades, social scientists have been responding to calls to improve understandings of the role played by context and culture on clinical practice in acute care environments.^1 2^ Social scientists have also been identified as critical contributors to public health emergencies, adding value by assessing social, economic and political factors as these shape responses to emerging public health crises across low-income and middle-income country (LMIC) and high-income country (HIC) contexts.^3 4^ A Wellcome Trust report recently recommended ‘fully integrating social science into epidemic preparedness and response’,^5^ with the World Health Organization (WHO) describing this integration as both a method to ‘build institutional and Member States buy-in and capacity’^6^ for health emergencies, and an essential component of the current ongoing global response to COVID-19.^7^ This paper describes the theoretical foundations, deployment and outcomes of an interdisciplinary team of social scientists formed from Organisational Sociologist (OS) ethnographers and Human Factors (HF) experts, working with Infection Prevention and Control (IPC) specialists and other clinicians as part of a Canadian province’s COVID-19 response.

Working in HICs, organisational sociologists and other ethnographers have provided nuanced analyses of how quality and safety policy is generated,^8^ interpreted^9^ and implemented^10^ inside the organisations of acute care. Specifically, ethnography^11^ and HF^12^ have been broadly employed to support quality improvement (QI) and patient safety in HIC clinical environments. Where ethnography is a fully social scientific discipline pursuing ‘thick descriptions’ of social action,^13^ HF is a specialty with roots in engineering, design and psychology that concentrates on identifying and remediating human limitations and characteristics as they affect the performance of tasks, processes and systems.^14 15^ HF incorporates knowledge from the biological and social sciences, with specialists in HF employing these approaches not just to study how humans use ‘anything and everything’, but to ease and optimise that use.^16^

The 2014 Ebola outbreak in West Africa focused both the ethnography and HF community’s attention on integration as a potential benefit to affected communities, and to response organisations themselves.^17 18^ Although there was an initial lack of clarity as to what OS, anthropologists and other ethnographers could contribute, discussions eventually led to the creation of innovative research platforms, including the Ebola Response Anthropology Platform (ERAP).^19 20^ Focused on how culture shapes the uptake of, or resistance to, public health policy, this research has tended to focus on LMIC contexts, or vulnerable populations in HIC contexts. The Ebola response similarly stimulated HF research and optimisation work with strides made in the effective use of personal protective equipment (PPE) and improvement of organisational responses.^21^ This applied research in the context of an outbreak saw IPC specialists—physicians and nurses with backgrounds in infectious diseases (ID) and limiting the transmission of pathogens—working alongside HF experts and clinicians to deliver practical recommendations aimed at optimising ease of use, workflow and safety.

Despite this progress, the simultaneous integration of OS, HF and IPC insights into organisations involved in epidemic preparedness and response was limited until the arrival of COVID-19. More importantly, response organisations tended to ‘slot’ social scientists into the role of cultural brokers, rather than direct contributors to health system response processes. The case study we report here illustrates a novel trend towards positioning interdisciplinary teams of these experts as ‘situated intervenors’ embedded in and directly contributing to health system response processes.

In this paper, we describe early progress and contributions made by a team of OS ethnographers, HF specialists and IPC experts carrying out ‘situated interventions’, which is to say, studying and feeding findings back into the health system in the HIC context of Alberta, Canada.^22^ Recognising that a ‘disease outbreak is no place to begin to negotiate disciplinary differences’,^3^ this multidisciplinary group was in contact well in advance of COVID-19. Our QI-focused ethnographers knew and had worked with our IPC professionals in the past, and indeed co-wrote the grant that would fund the present work. Similarly, our ID specialists had spent considerable time during the Ebola outbreak working alongside our HF colleagues, and recognised the added value of integrating OS, HF and IPC perspectives into clinical practice—particularly during health emergencies.^21 23^

Our work is grounded in the Wellcome Trust report’s observation that a ‘key aspect of saving lives during an infectious disease epidemic is the effective generation and use of contextual information and knowledge that can guide adaptive planning, agile decision-making and more effective interventions’.^5^ In this way, we are using our ongoing work as a case study, to describe the specifics of how health systems and emergency responders might leverage social science to improve the quality and safety of their pandemic responses and communications. The design and execution of our work, then, is a pragmatic effort to merge the traditions of public health focused and acute care focused social science.

## Background

Alberta is a landlocked province in the west of Canada. It is the nation’s fourth most populous jurisdiction and home to approximately 4.1 million residents spread over 640 000 km^2^. The province identified its first COVID-19 case on 5 March 2020, and, as of writing, has conducted over 500 000 tests, identified over 9100 cases and had 165 fatalities. Alberta’s population is concentrated in two major cities: Calgary (1.285 million) and Edmonton (972 000). As elsewhere in Canada, healthcare is delivered by the province, with Alberta having amalgamated what were nine regional health authorities in 2008 to create the province’s largest single employer: Alberta Health Services (AHS).

The social scientific team and work we describe below have been focused on understanding the preparation, communication and implementation of COVID-19 responses in the province’s public health, acute care and primary care sectors. All three areas of these have stronger or weaker ties to the central command structure of AHS. Thus, a study of how policies and communications are, or are not, flowing through and between the areas continues to generate real-time course correction material, and long-term learnings to shape future health emergency preparedness.

Our team secured funds through the Government of Canada’s COVID-19 Rapid Response Operational funding programme to use social science methods to describe the flow and implementation of policy in Alberta’s response.^24^ What follows details the theory behind the situated interventions we have been carrying out, as well as the deployment, and outcomes of our team as we have mobilised to address the pandemic.

## Research approach

### Theoretical foundation

Our team’s approach draws from Zuiderent-Jerak’s concept of generating ‘situated interventions’.^22^ Like Participatory Action Research (PAR)^25^ and Engaged Scholarship (ES),^26^ situated interventions seek to combine the work of producing scholarly understanding and changing practices. This approach stresses the production of an experimental space where the investigation of a concrete problem leads to the emergence of new ‘normativities’—senses of what is right and works, and what is wrong and does not work. Here, both practitioners and researchers engage with specific problems and co-produce new norms as they trade political and technical ideas. This entanglement transcends traditional dichotomies between: knowledge production and practice engagement^22^; research and quality improvement^22^; university and civil society^27^ and as discussed below, ‘insider’ and ‘outsider’ approaches to doing qualitative research. Rather than seeking to bridge or fill in gaps between these often dichotomised concepts, our work proceeds from the assumption that, as much as these might be analytically helpful distinctions, posing them as binary opposites does not contribute to nuanced, bi-directional learning. The situated interventions our team is undertaking are not just another form of implementation science in which prefixed norms or standards are imposed. Our research seeks to learn about, engage with, and work through the technical knowledge, operational concerns and political problems, of participants responding to the pandemic across Alberta’s health system.

Practically speaking, our multilevel approach to understanding the province’s COVID-19 response means we are learning about technical and social activity in a selection of more-or-less partitioned domains of health service provision: public health, acute care and primary care. We are then taking these learnings, not as established facts, but rather as new possibilities filtered through our own expertise, into adjacent domains. Here, we are engaging in the local politics of changing structures, processes and cultures, and improving quality not so much as advocates, but rather as experimentalists. While our team recognises our particular positionality, we also stress our fluid engagement across multiple groups and sites. For this reason, we see ‘situated interventions’ as describing our research activities and approach more accurately than other available concepts such as integrated Knowledge Translation (iKT), PAR or ES. Both the balance that is missing from PAR and ES, and the sociopolitical dimensions that are minimised in iKT, are captured in the self-reflexive, bi-directional learnings of the ‘situated interventions’ we are conducting. We are engaged with our participants, and negotiating new norms of activity alongside them.

### Deployment

Our team went into the field 6 days after our grant funding was announced and Alberta reported its first positive COVID-19 case.^28 29^ This meant that our ongoing research started at the moment Alberta began transitioning from preparedness to response modalities.

Our approach has been to engage with key informants and observe activities across public health, acute, and primary care settings. Specifically, we have been conducting interviews with a broad range of public health and clinical professionals to elicit their understandings of how information and policies are being sourced, vetted, interpreted, repackaged, retransmitted and implemented onto the operational front lines of acute and primary care. Our observations are focused on the flows of information and other human factors shaping the implementation of policies and protocols in both acute and primary care. Of particular interest are how AHS’ central, vertically integrated structure is, or is not, assisting with the adaptation of policy to local conditions and the sharing of those adaptations between local sites. To this end, we are gathering data on the challenges inherent in complying with public health and IPC guidelines as these constantly change.

Finally, we are not just observing, but facilitating preparedness simulations with primary care clinicians as they set up novel community-based clinics, or retro-fit existing facilities, to serve patients with COVID-19. These sessions see our ethnographic team members working alongside HF specialist colleagues and IPC experts, as well as primary care clinicians who are designing and implementing entirely new spaces. Our social scientific focus—beyond the technical work of building facilities that balance robust IPC processes with pragmatic physical and cultural realities—is again on understanding how high-level policies and priorities are interpreted through context and enacted at the clinical frontlines.

Across these deployments, we are positioned as a non-official group that happens to have connections to leadership in public health, primary and acute care clinical environments. As situated intervenors, we have been able to take up the role of ‘*alongsiders*’ rather than evaluative ‘outsiders’, or fully captured ‘insiders’.^30^ This ‘*alongsider*’ status, while challenging to maintain normally, has been relatively easy to accomplish given the level of organisational flux and participants’ appetites for implementation solutions that are both contextually congruent with local values, and compliant with broader directives. Our experience to date confirms that the changes and actions demanded of health leaders and frontline clinicians by the pandemic are occurring rapidly, and taking place within complex social and operational environments that are themselves changing in both predictable and unpredictable ways.^31^ Our situated interventions have focused on cross-referencing reactions to these predictable and unpredictable changes in different sectors, applying our expertise and knowledge to the challenges, and joining in local negotiations to prototype and pilot solutions. In this way, our ethnographic and HF observations and recommendations are focusing on better understanding the complexity of these environments and the perspectives of the people who move through them: doctors, nurses, reception and cleaning staff, patients and families.

### Data collection and iterative analysis

Based on the interdisciplinary nature of the team, we deploy mixed methods to gather our data. These are primarily based in observation, including semi-structured interviews with individual participants (n=55), virtual observations during emergency management group calls (n=43 hours) and in-person clinical shadowing in hospital units (n=14).

In addition, our joint OS/HF site visits involve in situ and virtual ‘walkthroughs’ guided by acute care and primary care clinicians. These sessions revolve around our team co-generating recommendations for improving the structures, processes and cultures that support safety. The sessions draw on both our expert knowledge and our multilevel view of policy and practice in other areas of the health system. After generating our recommendations, we have these reviewed by dedicated IPC experts before returning them to site-based or unit-based stakeholders for consideration and/or implementation. When those clinicians have encountered challenges in implementation, we have—as situated intervenors—become part of local efforts to create alliances and secure both knowledge and financial resources to address those challenges.

The core team has also convened focus groups with COVID-19 clinical working groups to discuss the role played by formal and informal structures, processes and communication pathways in creating and transmitting new policies and practices, as well as learnings from the first wave of the pandemic (n=2).^32^ In this way, we are integrating ourselves into ongoing QI processes that are activated before the emergency has been declared ‘over’.

The ethnographic data that have been collected during interviews, clinical shadowing and focus groups will continue to be iteratively analysed and re-coded by the core team. We use these data to cross-pollinate our recommendations and provide contextualised information to those who we are working and learning alongside. Our observations, interviews and site-based walkthroughs are ongoing across the province.

## Outcomes and findings

To date, we have conducted situated interventions to ameliorate communications across the public health, acute and primary care sectors of the province’s health system. Specifically, we have fed back, in real-time, emerging preparedness challenges as the acute care system has worked to operationalise constantly evolving public health policy. This has involved alerting central public health authorities to the need for integrated communication channels at the Zone Emergency Operations Centres that draw centralised communications across to departmental and unit leadership at specific clinical sites. Drawing public health policy makers’ attention to these challenges has resulted in a new emphasis on efforts to cascade high-level policy down through novel structures closer to local operational and clinical environments. Our primary care research has also identified a critical gap in IPC resources for community-based outpatient care. Out of our team’s efforts to remediate this gap—efforts that included leveraging our team’s access and understanding of the broader health system—a community of practice, focused on primary care IPC, has emerged.

In addition, at the clinical frontlines, we have catalysed the development of specific protocols for patient transfers between units and departments, based on ethnographic observations on unit floors and inputs from frontline care providers. Similarly, our ethnographic and HF walkthroughs have also supported COVID-19 designated inpatient units to improve their floor layouts, donning and doffing routines for PPE, and organise their patient transfer protocols between units. Our work has supported the sharing of existing clinical guidelines and procedural documents for COVID-19 care to sites beyond where they have originated, identifying potential efficiencies in how protocols can be transferred between hospitals, rather than re-invented. Our expanding connections to local, zonal and provincial stakeholders in primary, acute and public health environments—along with our willingness to join in the politics of transmitting knowledge that solves the problems of participants—mean we have been able to circulate and amplify these documents far beyond the sites in which they originate.

While much of the province’s response has been predicated on an infrastructure built from ‘lessons learnt’ from the 2009 H1N1 pandemic, it is clear that adjustments for the unique severity of COVID-19 are necessary. Our long-term work seeks to understand how these adjustments are both facilitated at the level of policy revision, protocol development and clinical practice. In addition to studying how old protocols are being repurposed, we are accelerating the adoption of rapid structure, process and culture improvements to prepare the healthcare system for a sustained burden of COVID-19 cases.

Similarly, we are working alongside primary care leaders to bridge gaps between their clinical practices and AHS’ COVID-19 advice and guidance. In this capacity, we have begun facilitating the introduction of training simulations targeting patient flow, COVID-19 care pathways and IPC into primary care settings. Finally, we have formalised our feedback by developing an online platform and curated repository for policy briefings, recommendations and stakeholder-informed advice that incorporates social scientific considerations of the outbreak’s cultural and political dimensions.^33^

As an emerging finding during our early research, we have discovered that ‘task shifting’ is a major component of pandemic health system responses worldwide. This phrase was originally understood as a key response and strategy at a global level to the AIDS crisis, whereby less specialised healthcare workers or community members would be able to support the delivery of healthcare through the redistribution of tasks.^34^ It is now used widely in global mental health to describe the provision of care ‘where there is no psychiatrist’.^35 36^

We have found that in the context of this pandemic, ‘task shifting’ and the rapid redistribution of clinical operations among a wide variety of medical personnel is a critical strategy. This strategy is continually deployed to shift human resources for healthcare workers across units and practice areas to surge where and when they may be needed. We recognise this continual redeployment, in other words, is not just a strategy for LMICs with limited resources in clinical settings, but for all countries dealing with the scarcities and system stresses introduced by the COVID-19 pandemic.

## Lessons learnt

We quickly recognised that triangulating disciplines and expertise within the team was essential for our research deployment. HF site visits in acute and primary care clinics were critical means to elicit ethnographic insights for OS ethnography team members. Likewise, our team’s sustained ethnographic observations have precipitated HF recommendations to improve unit layouts, communications and workflows. As an example, our HF team provided discrete recommendations to improve the organisation and layout of PPE on a COVID-19 designated hospital unit, and our OS/HF team, during independent observations, was able to describe and note how these changes were implemented on the floor and with the staff. Throughout the research, our work has relied on expert input from IPC clinical experts to ensure we are supporting frontline healthcare workers, staff and patients with appropriate recommendations. Each discipline uses different methodological approaches, which have synergised, and have bolstered mutual findings and discoveries.

Our team also relies on agility to keep pace with the rapid changes introduced by the COVID-19 response. Experts from acute care have had to pivot into providing support to primary care, and vice versa. Our anthropologist has had to become versed in IPC protocols and best practices, to support clinical environments and interpret ever-evolving guidelines and policies. Our clinicians have had to understand the differences between HF clinical walkthroughs and sustained ethnographic observation, and how best to support our team’s unique methodological hybridisation to achieve situated interventions. In doing so, we have been able to transport and share findings from distinct clinical and public health environments to support emerging best practices during the rapid changes in the COVID-19 response.

We have also found that our breadth of perspectives across public health, acute care and primary care are critical in triangulating our findings and developing a ‘whole system’ understanding of the response, its communication pathways and the impact these have on clinical practice and the implementation of policy. Our interviews with key stakeholders involved in the public health emergency have supported a better understanding of clinical practices on the frontlines, and vice versa. In this sense, our work as social scientists has been focused on ‘bottom-up’ analysis, and on bi-directional processes and communications across normally discrete segments of the health system as they have rapidly adjusted to the pandemic.^3^

## Key challenges

Given the speed of change and the rapid appearance of novel emergency management and response groups, our team has found it difficult to remain consistently involved and engaged across multiple environments in the health system. Due to our position as ‘*alongsiders*’, our access to relevant individual or group stakeholders has not been guaranteed and must be negotiated on a case-by-case or site-by-site basis. Our team is also reliant on pre-existing relationships with hospital units, site leadership or administrators to conduct observations and have any resulting recommendations considered or implemented. We rely on goodwill and buy-in from individual participants who are willing to take time out of their busy schedules during the pandemic to reflect on their own work and allow our team access to their units, hospitals, clinics or offices. Additionally, because many higher-level stakeholder groups have been unavailable to engage with our team, and our team was not officially integrated into the provincial government response, we have thus far struggled to recruit participants at these levels and so have a diminished capacity to fully represent the dynamics of the pandemic response. While on the one hand, our situated intervention approach to working ‘alongside’ participants allows us to triangulate our observations across multiple areas, our lack of official designation means that while some of our findings have been explicitly integrated into the province’s formal health system responses,^37^ many have not made it beyond the local areas where they have been generated.

Operational approvals to conduct our research within the health system were also significantly delayed because the pre-established approval process did not accommodate our innovative methodological approaches, align with social scientific methodologies more generally or offer the flexibility required to pivot rapidly in the face of changing pandemic conditions. The approval process, required by AHS to access acute care and public health facilities, is predicated on experimental or clinical trials research, rather than on non-invasive ‘*alongsider*’ situated interventions. This is likely a barrier that other social scientists will encounter as they attempt to integrate their research into clinical operational environments. We recommend that social scientific, interdisciplinary research teams involved in pandemic responses should engage early with system stakeholders to adapt or preformulate operational approval mechanisms that accommodate and expedite real-time social scientific or observational research integration into public health responses.

## Conclusion

Our work interviewing and observing public health, acute and primary care personnel has led to social scientific insights that will be published according to traditional academic timelines, and to situated interventions that deliver substantive feedback directly to the province’s health system. Highlighting communication and implementation gaps in the existing response, we have blended OS ethnographic techniques with the methods and expertise of both HF, IPC and a range of clinical practices.

Where much of the literature in the epidemic space situates social scientists as cultural brokers,^38 39^ we have instead adopted a proactive approach to integration. Rather than seeking to translate or provide context for a community’s hesitancies and health-seeking behaviours, to increase public acceptance of public health interventions, or to provide critical commentary on the politics of epidemic responses,^40^ we are deploying ethnography, HF and IPC expertise alongside public health professionals as well as acute and primary care clinicians. Our aim in this is to improve the system’s ability to understand and communicate with itself in the context of the response. As this implies, our focus, along with the insights we are feeding back into the system, are centred on communications, which have been identified as a critical priority during health emergencies and epidemics.^41^ This shift in emphasis highlights a unique space for social scientists in a pandemic, integrating them into the response as situated intervenors of emergency communications and best practices between stakeholders. This is occurring as those stakeholders deal with multiple competing priorities; a lack of—or contradictory—clinical and ethical knowledge; current and possible operational pressures; resource inadequacies and efforts to maintain professional boundaries and scopes of practice.^42 43^

The theoretical foundation, deployment and outcomes of our team offer a critical example of how social scientists and their methods can be leveraged to improve an evolving pandemic response and to support long-term epidemic and pandemic preparedness. Although additional findings are forthcoming, this swift assembly of a team of social scientific ‘*alongsiders’* aimed at delivering situated interventions in a highly fluid and rapidly changing environment has already led to discrete recommendations and related system reforms during the COVID-19 pandemic in Alberta.
